# Agreement Between a Large Language Model and the Revised Geneva Score Across Synthetic Patient Profiles: A Computational Concordance Study

**DOI:** 10.7759/cureus.119442

**Published:** 2026-11-10

**Authors:** Thomas F Heston, Grace Hwang

**Affiliations:** 1 Medical Education and Clinical Sciences, Washington State University, Spokane, USA; 2 Family Medicine, University of Washington, Spokane, USA; 3 College of Engineering, University of Washington, Seattle, USA

**Keywords:** clinical decision rules, large language models, pulmonary embolism, reproducibility, revised geneva score, risk stratification

## Abstract

Background: This study examined how a frontier large language model (LLM) evaluating patient profiles at risk of pulmonary embolism categorizes risk compared with the revised Geneva score. The study also asked whether the LLM, given the score's nine components but not the score's name, would assign each component its published point value.

Methods: Claude Opus 4.8 (Anthropic, San Francisco, CA, USA) was compared with the revised Geneva score. All 384 possible patient profiles were enumerated. The LLM was asked to weight the score's components, then classify every profile as low, intermediate, or high probability. The revised Geneva score was never named. Risk classification ran twice under each of two wordings: all findings named present or absent, or only present findings listed. Responses were also examined for the score named and the total computed.

Results: The model gave every component the same point value as the revised Geneva score. Risk classification nonetheless diverged, and divergence tracked prompt wording. Agreement with the score averaged 41.7% under present-and-absent prompting and 62.6% under present-only. Of the 217 profiles the revised Geneva score rated high risk, the present-and-absent prompt called 24.2% low risk, versus 1.4% for the present-only prompt. When the LLM gave its reasoning for risk assignment, it named a Geneva score 91% of the time. Among responses that showed reasoning, named a Geneva score but not Wells, and stated a total, the total matched the revised Geneva score in 104/197 (52.8%) under present-and-absent and 89/97 (91.8%) under present-only. Most classifications had no assessable total.

Conclusions: This study assessed agreement with the revised Geneva score in simulated patients; diagnostic accuracy and patient outcomes were not assessed. Claude Opus 4.8 reproduced the score’s point values exactly, yet its risk classifications often disagreed with the score and varied depending on how the patient was described in the prompt.

## Introduction

Large language models (LLMs) are increasingly proposed as point-of-care aids and have shown potential to assist physicians in clinical diagnostics, triage, and risk stratification [1-3]. Much of the evidence comes from performance on medical licensing examinations, which does not necessarily translate into reliable clinical decision-making. A systematic review of 39 benchmarks found performance substantially lower on practice-based than on knowledge-based tasks [4]. In a simulated clinical workflow, LLMs deviated from diagnostic and treatment guidelines, performed worse than physicians, and proved sensitive to the quantity and order of the information presented [5]. A meta-analysis of 83 studies found generative artificial intelligence significantly less accurate than expert physicians (difference 15.8%, P = .007) [6].

A model can often state a validated clinical decision rule correctly when asked. Stating a rule and executing it on a specific patient, however, are different acts. Unassisted ChatGPT produced incorrect responses in about one-third of medical calculation trials across 48 tasks [7]; on MedCalc-Bench, a benchmark of over 1,000 cases across 55 medical calculation tasks, model failures spanned entity extraction, use of the correct equation, and arithmetic computation [8]; and step-wise re-evaluation shows final-answer metrics can mask formula and extraction errors [9]. Those studies instruct the model which score to compute. What happens when a model is not told and must decide for itself has not been separated from what happens when it is told.

Venous thromboembolism is the third most frequent acute cardiovascular syndrome worldwide, behind myocardial infarction and stroke [10]. Of an estimated 370,000 venous thromboembolism-related deaths across six European countries in a single year, 34% were sudden fatal pulmonary embolism and only 7% were diagnosed antemortem [11]. Missed diagnosis is not the only cost. Over-triage generates unnecessary imaging and consumes hospital capacity. Overdiagnosis of subsegmental pulmonary embolism has been described as a countervailing tendency of the modern diagnostic era [10].

The revised Geneva score is a validated clinical rule that estimates the likelihood of pulmonary embolism from eight clinical variables, with integer weights, and applies fixed thresholds to categorize risk as low, intermediate, or high [12]. This diagnostic pretest-probability classification differs from prognostic stratification after confirmed pulmonary embolism, where established tools can assign different categories to the same patients [13]. The widely used Wells rule includes the physician's judgment of whether an alternative diagnosis is more likely, a criterion that cannot be standardized; the revised Geneva score’s eight variables are objective and independent of clinical judgment [14,15]. Its accuracy is nonetheless similar to the Wells rule [14-16]. This makes it a computable reference standard against which model classifications can be compared. Its input space is also small, comprising eight variables with fixed weights and cutoffs, so every possible profile can be enumerated rather than just sampled.

Existing literature has evaluated LLM performance in triage, diagnosis, and stratification by comparing model recommendations with human triage staff decisions [1], guideline-based answers [2], or other reference standards [3,17]. These studies evaluate the final output without distinguishing the model's knowledge of a rule, its decision to use that rule, and its execution of it. The present study evaluates those three where observable. It asks whether the model reproduces the established rule’s weights; how often its classifications agree with the rule; whether its explanations name the rule and report the correct total; and whether this holds when the same patient is described differently.

## Materials and methods

Study design

This was a computational concordance study. A frontier LLM was compared with the revised Geneva score on two tasks: reproducing the score's component weights, and classifying every profile in the score's complete input space. All patient profiles were simulated using synthetic combinations of the score's input variables, so institutional review board approval and informed consent were not required.

Study setting and population

The reference standard was the revised Geneva score [12], which assigns integer points to eight objective findings and sorts patients into three risk categories using fixed cutoffs: low (0 to 3 points), intermediate (4 to 10 points), and high (11 or more points). The findings and their point values are age over 65 (1), previous deep vein thrombosis or pulmonary embolism (3), surgery or lower-limb fracture within one month (2), active malignancy (2), unilateral lower-limb pain (3), hemoptysis (2), heart rate 75 to 94 (3) or 95 or greater (5), and pain on lower-limb deep venous palpation with unilateral edema (4).

The score has seven binary findings and three heart-rate levels, so its entire input space is 2^7 × 3, or 384 profiles. The score classifies 11 of these as low risk, 156 as intermediate, and 217 as high.

Study protocol

A frontier LLM (Claude Opus 4.8, model identifier claude-opus-4-8; Anthropic, san Francisco, CA, USA) was queried between June and August 2026 through its application programming interface. Sampling temperature could not be fixed as the parameter is deprecated for this model. Each query was single-turn, with no system prompt and no conversation history carried between profiles; the maximum output length was 2,000 tokens, and no seed was specified. The model's training corpus and knowledge cutoff are not publicly documented, so exposure to the revised Geneva score could not be confirmed.

The weighting prompt listed the components of the revised Geneva score, without naming it, and asked for a numeric weight for each component. It was repeated 10 times. A reworded version in lay terms and a different order was repeated 10 times as a paraphrase check. Thresholds were not elicited.

Each of the 384 profiles was presented as a patient with suspected pulmonary embolism; age and heart rate were given as values in the relevant band (48 or 72 years; 68, 88, or 104 beats per minute). The model was asked for one word: low, intermediate, or high. The revised Geneva score was never named, and the model was never told to apply it. Both wordings gave age and heart rate as numeric values and differed in the remaining findings: the present-and-absent wording stated each component as present or absent, while the present-only wording listed only those present, and stated in one line that anything unlisted was absent. Each wording ran over all 384 profiles twice, giving 1,536 queries.

The present-only wording was written after the present-and-absent wording showed downward discordance, to test whether the individually listed negatives were driving it; this comparison is exploratory. Runs were collected June 29, July 5 (two runs), and August 10, 2026.

Measurements

Weight responses were read from the nine numbered lines the prompt required; all 20 complied with that format. Each response yielded nine values, compared against the published revised Geneva weights. The reworded prompt listed the components in a different order, so its values were remapped to the original ordering before comparison.

The model frequently ignored the single-word instruction in the classification prompt and returned a worked calculation ending in a category. Each answer was therefore taken by scanning from the last line up and using the first line that reduced to exactly low, intermediate, or high after removing non-alphabetic characters. The rule was fixed after inspecting the response formats in the data, then applied uniformly to all 1,536 responses; it resolved every one, with no exclusions or subjective judgment.

Two further quantities were extracted from classification responses that showed reasoning: whether the response named a Geneva score, the Wells rule, both, or neither; and, for responses naming a Geneva score but not Wells, the numeric total stated, taken from the final line containing the word total and truncated before any interpretive clause. Totals were compared with the profile's revised Geneva score. Responses naming both scores were excluded, since the total cannot be attributed. Extraction was checked against the printed total lines.

Data analysis

Concordance with the revised Geneva score was summarized by raw agreement and quadratic-weighted kappa. Kappa was primary because a two-category miss is worse than a one-category miss, but it is sensitive to the marginals, so raw agreement and the full confusion tables are reported alongside it. Because every profile was tested rather than sampled, there is no sampling variability over patients; the remaining variability is the model's own, which two runs can only crudely bound, so no interval estimate is offered. Because the 384 profiles are an enumeration rather than an independent sample, comparisons are reported descriptively, without significance tests.

Two within-model tests were run. First, for every ordered pair in which one profile carried all of another's findings plus at least one more and no lower heart-rate band, the richer profile was required to be classified no lower than the poorer; any rule with non-negative weights satisfies this, whatever the weights, so violations cannot be attributed to different weighting. Second, within each stratum of identical revised Geneva score, possible because the weights differ, every pair of profiles differing in the number of absent findings was compared and tallied by whether the profile with more absent findings received the lower category, the higher, or the same.

Analysis was performed in Python using pandas. Quadratic-weighted kappa was computed from first principles rather than from a library function. The deposited analysis script reproduces all reported values from the archived raw responses. The study comprised 1,556 queries: 1,536 classification and 20 weight-elicitation. Analysis scripts, prompt texts, raw responses, and derived data are archived on Zenodo (https://doi.org/10.5281/zenodo.21866462).

Reporting follows the Transparent Reporting of a multivariable prediction model for Individual Prognosis Or Diagnosis for large language models (TRIPOD-LLM) guideline [18]; the completed checklist is available in the Zenodo repository (https://doi.org/10.5281/zenodo.21866462).

During the preparation of this work, the authors used Claude Opus 5 (Anthropic) for assistance with language editing, coding, and formatting. The authors reviewed and edited the output as needed and take full responsibility for the content of the published article.

## Results

Weight assignment

On the weighting prompt, the model returned the exact published revised Geneva weights on all 10 repetitions, with no variation across queries: 1, 3, 2, 2, 3, 2, 3, 5, 4, in the order the findings were listed. The reworded version, which listed the same nine components in lay terms and in a different order, returned the same weights on all 10 repetitions once remapped to the original ordering.

Risk classification concordance

Both classification prompts requested a single-word risk classification: low, intermediate, or high. Both prompts gave age and heart rate as numeric values. The present-and-absent prompt stated each of the six remaining findings as present or absent; the present-only prompt listed only those present. The model showed its reasoning instead of the single word requested in 137/384 (36%, first run) and 147/384 (38%, second run) of responses to the present-and-absent prompt, and in 56/384 (15%, first run) and 52/384 (14%, second run) to the present-only prompt.

Risk classification diverged from the reference standard, and the divergence depended on prompt wording. Under present-and-absent, raw agreement was 0.438 in the first run and 0.396 in the second (mean 41.7%), with quadratic-weighted kappa 0.312 and 0.311 (mean 0.31). Under present-only, raw agreement was 0.617 and 0.635 (mean 62.6%), with kappa 0.534 and 0.545 (mean 0.54).

These results describe the observed runs, not the model’s general performance. Run 1 and run 2 assigned the same category to 282/384 profiles (73.4%, κ 0.755) under present-and-absent and 296/384 (77.1%, κ 0.733) under present-only; 102 (26.6%) and 88 (22.9%) profiles changed category between runs.

Of the 217 profiles the revised Geneva score classified as high risk, 52 (first run) and 53 (second run) were classified as low risk under present-and-absent (mean 24.2%), compared with four (first run) and two (second run) under present-only (mean 1.4%). When the model disagreed with the revised Geneva score, it nearly always assigned a lower category (Tables 1, 2), reflecting disagreement with the score rather than demonstrated clinical under-triage.

**Table 1:** Classification of the 384 enumerated revised Geneva profiles under the present-and-absent prompt.

Revised Geneva Category	Model: low (run 1 / run 2)	Model: intermediate (run 1 / run 2)	Model: high (run 1 / run 2)	Total
Revised Geneva: low	11 / 11	0 / 0	0 / 0	11
Revised Geneva: intermediate	91 / 103	64 / 53	1 / 0	156
Revised Geneva: high	52 / 53	72 / 76	93 / 88	217
Total	154 / 167	136 / 129	94 / 88	384

**Table 2:** Classification of the 384 enumerated revised Geneva profiles under the present-only prompt.

Revised Geneva Category	Model: low (run 1 / run 2)	Model: intermediate (run 1 / run 2)	Model: high (run 1 / run 2)	Total
Revised Geneva: low	46336	0 / 1	0 / 0	11
Revised Geneva: intermediate	45 / 38	91 / 103	20 / 15	156
Revised Geneva: high	46114	78 / 84	135 / 131	217
Total	60 / 50	169 / 188	155 / 146	384

The model's agreement with itself from run 1 to run 2 exceeded its agreement with the revised Geneva score under both wordings, by 30 to 34 percentage points under present-and-absent and 14 to 15 under present-only (Table 3).

**Table 3:** Agreement of the model with itself and with the revised Geneva score.

Wording	Run 1 vs run 2	vs Geneva, run 1	vs Geneva, run 2
Present-and-absent	0.734 (κ 0.755)	0.438 (κ 0.312)	0.396 (κ 0.311)
Present-only	0.771 (κ 0.733)	0.617 (κ 0.534)	0.635 (κ 0.545)

Rule selection versus computation

Among the responses that showed reasoning, the model named a Geneva score in 89% to 93% of cases in all four runs (mean 91%), with no meaningful difference between wordings; a minority named the Wells rule instead, in 15 and 12 responses under present-and-absent and four and five under present-only. The wording change affected how often stated totals matched the score. Among responses that named a Geneva score, did not name the Wells rule, and stated a numeric total, the total matched the profile's published revised Geneva score in 57 of 96 (59%) and 47 of 101 (47%) under present-and-absent, pooled 104 of 197 (52.8%), against 46 of 52 (88%) and 43 of 45 (96%) under present-only, pooled 89 of 97 (91.8%). Of the totals that did not match the revised Geneva score, 16 of 93 under present-and-absent and one of eight under present-only matched the simplified Geneva score. The other 1,242 responses had no attributable total and were unassessable for calculation accuracy.

Within-model tests

The model contradicted itself in ways no set of weights can explain. Of the 12,738 ordered profile pairs in which one profile carried every finding of the other plus at least one more, and no lower heart-rate band, the model assigned the richer profile a lower category in 379 pairs (3.0%) and 323 (2.5%) under present-and-absent and in 423 (3.3%) and 299 (2.3%) under present-only. Adding a risk factor lowered the model's stated probability in 2% to 3% of these comparisons. Separately, of the 3,114 within-stratum pairs of profiles with identical revised Geneva score but differing numbers of absent findings, the profile with more absent findings was assigned the lower category more often than the higher in every run: 1,099 to 673 with 1,342 ties and 1,063 to 633 with 1,418 ties under present-and-absent, and 1,074 to 459 with 1,581 ties and 1,063 to 349 with 1,702 ties under present-only. The imbalance appeared under both wordings.

## Discussion

A frontier LLM (Claude Opus 4.8) reproduced the revised Geneva score's exact weights on demand. It named a Geneva score of its own accord when asked for a clinical probability, even though it was not told which rule to use. Among responses naming a Geneva score without Wells and stating a total, the totals often did not match the revised Geneva score; classifications also diverged under both wordings. Knowing the rule and choosing to use it did not mean the model could run it. Perfect weight recall looks like memorization, but recall was not what failed here.

The LLM's failure was wording-dependent, and stated totals and classification moved together. When presented with only the risk factors present, while stating all others were negative, the model’s stated total matched the published score correctly in 92% of scored responses and agreed with the score on 63% of profiles. But when prompted with both present and absent risk factors, matching totals fell to 53% and agreement to 42%. The rate at which it reached a Geneva score barely moved, staying between 89% and 93% across all four runs. The present-and-absent wording was associated with a lower proportion of reported totals matching the revised Geneva score among eligible responses, while the model named a Geneva score at a similar rate among responses that showed reasoning, consistent with failure modes reported for instructed medical calculation [8,9]. Accuracy is known to degrade with score complexity when the model is told which score to use [19]; here it degraded with nothing more than changing how the patient was described.

Rule divergence is not a coherent explanation. Across all four runs, 36 responses applied one alternative, naming the Wells rule, so these account for a small share of the discordance. In addition, the model's own orderings were internally inconsistent. Any rule whose findings only add points must rate a patient with more findings at least as high as a patient with fewer, but the model violated this in 2.3% to 3.3% of strictly dominating pairs. That rate is low and does not by itself explain the rule discordance.

We consider explicitly listing negative risk factors the most likely primary cause of the wording effect, although prompt length, information density, and presentation may also have contributed; their individual contributions were not isolated. Within strata of identical revised Geneva score, profiles with more absent findings drew the lower category more often in every run, although such profiles also differ in which findings are present. This finding supports previous research showing that negation handling is a recognized difficulty for clinical language systems [20].

The clinical implication is narrow but concrete. A model's ability to recite a score's weights predicts neither correct computation nor matching classification, and the model gave no signal of difficulty when its stated total was wrong, consistent with previous work showing that language models answer confidently even when no correct option is available [21], and remain overconfident while underperforming physicians on atypical presentations [22]. The model performed best when the case was presented as a compact list of positive findings, without mentioning specific risk factors that were absent.

The model ignored an explicit single-word output instruction in 36% and 38% of present-and-absent queries, indicating a pattern of noncompliance with direct instructions. While prompt engineering may improve LLM responses [23], noncompliance with explicit output instructions is already documented. One study found that GPT-4 followed every verifiable instruction in a prompt 78% of the time, failing at least one instruction on the remaining 22% [24]. Another study of structured electronic health record queries found compliance ranging from 99.6% for GPT-4o and Qwen-2.5 down to 0.4% for Llama-3.1-8B, depending on model and task [25]. Noncompliance with prompt instructions could have important consequences in high-stakes clinical settings.

Limitations

This study evaluated one LLM and one scoring system, limiting generalizability. However, testing all 384 combinations ensured complete coverage of the revised Geneva score’s defined input space. The two ages and three heart rates represented its categorical inputs. Furthermore, preliminary evidence suggests longitudinal convergence across model families in at least some dimensions [26]. The revised Geneva score is a reference standard for risk stratification, not diagnostic ground truth; furthermore, no patient outcomes were collected; therefore, discordant classifications cannot be ranked by clinical accuracy. However, the score has been externally validated and incorporated into international clinical guidelines, supporting its use as the reference standard for risk stratification [10,14-16]. Furthermore, this limitation does not affect the reported-total comparison, because each stated total can be compared directly with the published score. Uniform enumeration yielded 217 high-risk profiles, so aggregate agreement is not weighted to clinical prevalence; however, it ensured complete testing of the score’s input space. Whether these findings extend to other clinical scoring systems is a hypothesis; replication across other models and scored rules is the natural next study.

## Conclusions

Reciting a validated clinical decision rule, choosing to use it, and computing it correctly can differ, and a frontier LLM (Claude Opus 4.8) succeeded at the first two but applied the rule inconsistently, depending on prompt wording. In this synthetic benchmark, the model reproduced the revised Geneva score’s published weights exactly and named a Geneva score unprompted in most responses that showed reasoning, yet its classification concordance and stated totals varied with how the same patients were described. Concordance with a validated rule should be demonstrated on the intended input format, not inferred from a model’s ability to state the rule, and independently validated before clinical use.
